# Understanding policy alignment in addressing hydrological hazards in the Niger Delta region, Nigeria

**DOI:** 10.1371/journal.pone.0345583

**Published:** 2026-03-25

**Authors:** Obroma O. Agumagu, Robert Marchant, Lindsay C. Stringer

**Affiliations:** Department of Environment and Geography, Wentworth Way, University of York, Heslington, York, North Yorkshire, United Kingdom; Sathyambama Institute of Science and Technology: Sathyabama Institute of Science and Technology (Deemed to be University), INDIA

## Abstract

Combating climate change, flood risk, managing land use, and developmental change need cohesive policy action. Analysing policies is essential to assess whether there is a coherent approach where various sectors support each other, or at least do not conflict. This study analyses policy documents addressing hydrological hazards in the Niger Delta region, Nigeria using Qualitative Document Analysis, content analysis, keyword analysis and frequency counts. These insights were used to examine: 1) the types of hydrological hazards and their drivers recognised in six national policy documents across environment, climate change, agriculture, water, forest, and petroleum policy sectors, 2) the measures outlined to reduce the risks from these hazards, and 3), how well aligned the measures are across sectors for management of the Niger Delta region. Results reveal that the policies across the Environment and Climate Change directly address hydrological hazards and their climatic drivers. In contrast, Agriculture, Water, and Forest policies demonstrate sector-specific approaches, while the Petroleum policy stands out for its very limited consideration of hydrological hazards and their drivers. Hydrological hazards were considered as high rainfall, river floods, sea level rise, storm surges, and warming trends as key hydrological hazards. Climate variability and human activity arising from urbanisation, deforestation, industrialisation, agriculture, and population are identified as drivers that can exacerbate the hazards and their impacts. Measures across the policies consider flood defence structures, preparing comprehensive hazard maps and vulnerability analysis to strengthen smart water management to reduce risk and build adaptive capacity across the region. Overall alignment of the six national policies is found to be low, indicating limited attention to the interactions between sectors and stakeholders. This pattern indicates horizontal misalignment. For Nigeria to better manage climate change impacts, all hydrological hazards and their drivers must be recognised. There is a need for the policy framework to be more joined-up so that a multi-sector approach can reduce risks from hydrological hazards.

## 1. Introduction

Governments worldwide have begun implementing measures to address the causes and impacts of climate change. Despite the commitments made under the 2015 Paris Agreement, global progress remains insufficient. The Intergovernmental Panel on Climate Change (IPCC) has highlighted this inadequate progress, emphasizing the urgency of the challenge for nations across the Global South [1]. The consequences of climate change are particularly acute in Africa due to the continent’s high dependence on the environment for subsistence, limited adaptive capacity, widespread poverty, and rapid population growth [2]. Furthermore, Africa faces significant climate data limitations and inequities in research funding and leadership, which further constrain its adaptive capacity [3]. Simultaneously, public climate literacy remains low; analysis of Africa’s largest representative public opinion survey indicates that climate change literacy ranges from 23% to 66% across 33 countries, with even greater subnational variation (e.g., 5–71% among Nigerian states) [4].

These deficiencies are highly problematic, as African nations, including Nigeria, are increasingly confronted by climate-related hazards that cause severe socio-economic disruption, loss of life, and damage to livelihoods, infrastructure, and the environment [5]. Many of these events are hydrological hazards extreme events associated with the occurrence, movement, and distribution of water, such as floods, droughts, high rainfall, storm surges, and rising sea levels [6].

The risk levels associated with hydrological processes are determined by their frequency, severity, and the extent of the resulting damage. Mitigating these risks, particularly in coastal regions like Nigeria’s Niger Delta Region (NDR), requires informed planning and the utilization of decision-support systems that account for the interplay between risks across various sectors [7]. Addressing hydrological hazards and building resilience requires a cooperative framework that facilitates connections between the environment, local communities, and diverse economic sectors [8]. These priorities are underscored by international initiatives, including the Sendai Framework for Disaster Risk Reduction, the Paris Agreement, and the Sustainable Development Goals (SDGs). While experts emphasize the urgency of impact assessments and probabilistic scenario analyses to understand risks, policy alignment is equally essential to ensure that sectors collaborate effectively to reduce impacts when extreme events occur [9].

This study recognizes that policies addressing climate change and development in Nigeria’s NDR must present a unified front and forge critical connections across government agencies, acknowledging that climate change is a cross-cutting issue. Such an approach enables the integration of climate risk reduction into national sectoral policies [10]. Indeed, policy analysis is increasingly vital for addressing climate challenges while balancing societal demands [11]. It clarifies the theoretical operation of policies and provides legislators with options to enhance implementation [12,13]. Furthermore, policy analysis provides the evidence base necessary to secure resources for climate adaptation and mitigation. By identifying collective policy goals, scholars can examine how alignment influences national development [14]. For example, policies promoting renewable energy should be coherent with land-use policies that protect natural habitats like mangroves, which serve as vital buffers against storm surges and flooding.

Alignment is defined as the degree to which connections exist between climate adaptation and risk management policies to provide a basis for coherence [15,16]. Although alignment is frequently cited as a priority, it has rarely been the centerpiece of implementation studies [17]. Nevertheless, national-level alignment can enhance efficiency in reducing exposure, sensitivity, and vulnerability across key sectors. Understanding how national policies incorporate hydrological hazards into these sectors is vital to assessing their contribution to national resilience.

**Fig 1:**
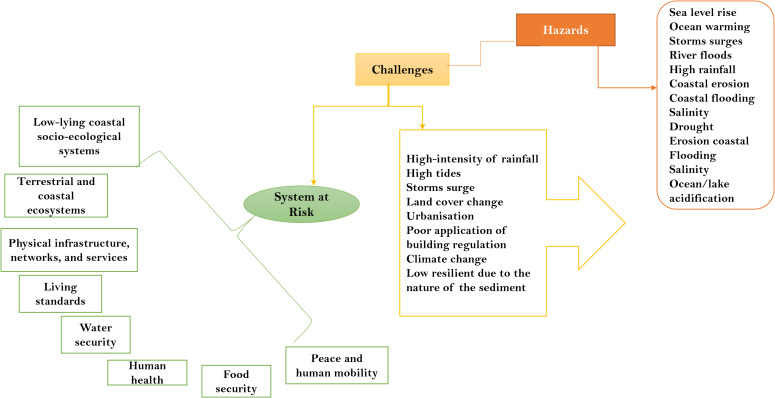
Climate hazards and the eight key risk sectors. The colours in green represent the key risk sectors, while the colours in yellow represent the challenges, and the colours in pink represent the hazards, which are adopted from [18].

The IPCC’s Sixth Assessment Report (2022) identifies climate hazards as representative key risks affecting low-lying coastal systems, as well as terrestrial and ocean ecosystems (Fig 1) [18].

These systems are evaluated here in the context of hydrological risks in the NDR across the environment, climate change, agriculture, water, forest, and petroleum sectors. Currently, research is lacking regarding how consistently different policies identify hydrological hazards and their drivers, and the extent to which strategies are coherent across key risk sectors. This paper aims to evaluate how hydrological hazards are considered across major policies and the degree to which these policies are aligned. Specifically, it examines: 1) the types of hydrological hazards and drivers identified in six Nigerian national policy documents; 2) the measures proposed to reduce these risks; and 3) the degree of alignment across sectors. While these policies are formulated at the national level, the focus on hydrological risks is of paramount importance to the NDR.

## 2. Climate change policy context in Nigeria

Nigeria signed the Paris Agreement in September 2016 and ratified it in March 2017, committing to significant reductions in greenhouse gas emissions. In its Nationally Determined Contribution (NDC), the country pledged an unconditional 20% reduction in “Business as Usual” emissions by 2030, with a conditional commitment of 47% contingent upon international financial assistance, technology transfer, and capacity building [19]. Efforts to achieve the 47% reduction focus heavily on renewable energy development and forest improvement. Consequently, Nigeria recognizes the imperative of integrating mitigation and adaptation into development projects to reduce the nation’s susceptibility to climate change [20]; in the Niger Delta Region (NDR), this integration is particularly critical given the prevalence of hydrological hazards.

Although Nigeria submitted its First National Communication to the UNFCCC in 2003, followed by the second in 2014 and the third in 2020 [19], the instrumental and legislative support required to align these commitments with developmental goals, especially in coastal regions, remains elusive [21,22]. Furthermore, responses aimed at mitigating and adapting to hydrological hazards have been characterized as slow [23]. The National Policy on Climate Change, adopted in 2021, targets several key areas: ending gas flaring by 2030, deploying 13GW of off-grid solar PV, improving energy efficiency by 2% annually, shifting transport from private cars to buses, enhancing electricity grid performance, and promoting climate-smart agriculture and reforestation [24]. This policy is intended to align with and support existing national adaptation efforts [25], including:

- The National Policy on Drought and Desertification
- The Drought Preparedness Plan (2007)
- The National Policy on Erosion, Flood Control and Coastal Zone Management (2005)
- The National Forest Policy (2006)
- The National Biodiversity Strategy and Action Plan (2004)

These national mandates must also be recognized in state-level decision-making. For example, climate change adaptation needs to be integrated into the Niger Delta Development Plan and other existing policies at the Delta scale [26].

Sustainable risk management relies on robust governance, where legal, policy, and institutional arrangements are viewed as shared responsibilities involving all relevant stakeholders and sectors [27]. However, Nigeria continues to face challenges in demonstrating how climate mitigation, adaptation, and development priorities can be cohesively integrated into national planning [28].

Against this background, this research analyses national policy documents covering the environment, climate change, agriculture, forest, water, and petroleum sectors, while considering the barriers to their implementation in the NDR. These sectors are prioritized because they directly target risks to human populations and are highly exposed to climate change. The analytical approach recognizes the multi-faceted and interlinked nature of sustainable development, which requires interventions to be tackled simultaneously through a coordinated approach [29].

Particular consideration is given to the NDR due to its coastal location and extreme exposure to diverse hydrological hazards. To date, policies in this region have been largely ineffective in achieving sustainable economic growth or development [30]. Access to funding, knowledge, capability, information, and government and private sector support has largely been lacking [31], hindering the region’s ability to confront contemporary environmental problems. Additional challenges include overlapping mandates among government agencies, regional insecurity, and the geographic remoteness of many coastal communities [32].

**Fig 2:**
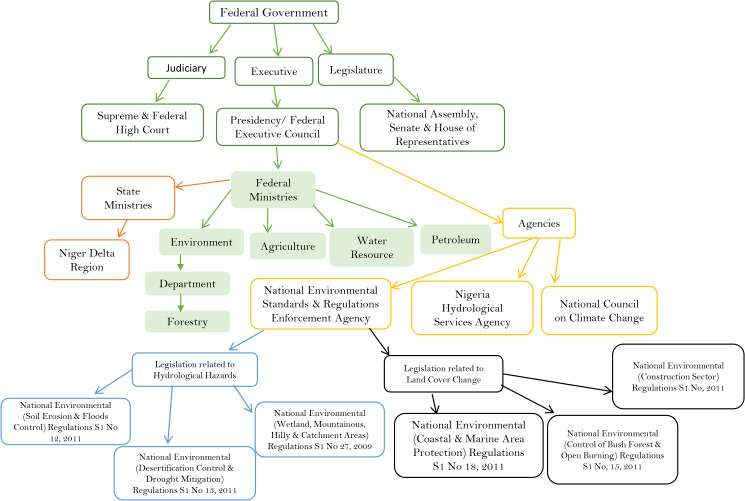
The organisation of Nigeria’s governing structure and a representation of legislation, laws, and standards in Nigeria dealing with environmental issues. Boxes outlined in dark green represent Nigeria’s governing structure, and sectoral ministries and departments are in lighter green. Yellow boxes represent the agencies, while the boxes outlined in blue represent regulations related to hydrological hazards. Boxes outlined in black represent regulations related to land cove.

Nigeria possesses multiple policies capable of addressing environmental management. The legislature holds the authority to enact, amend, and repeal legislation, serving as the legal foundation for all policy formulation. Environmental governance operates across national, state, and local levels, with policy options cutting across these layers. The Nigerian Parliament is bicameral, consisting of the Senate and the House of Representatives [19]. Together with the Executive, they have formulated numerous laws and regulations specifically targeting the NDR. Fig 2 illustrates Nigeria’s governing structure and the diverse legislation identifying climatic impacts and drivers relevant to the key risk sectors identified by the IPCC (Fig 1). These key risks were selected because they are projected to become increasingly severe as climate change intensifies [18]. For example, the risk to low-lying coastal socio-ecological systems encompasses the physical, human, and ecological components of those systems. Laws in Nigeria are enacted to address specific environmental issues, including hydrological hazards (Fig 2), and the implementation of these policies is managed by designated Ministry Department Agencies (MDAs).

## 3. Policies in the Niger Delta region

Given that the Niger Delta contributes substantially to Nigeria’s GDP [33], and it is exposed to many hydrological hazards, climate change presents a major challenge with impacts across the region. Policy analysis in response to hydrological hazards in the NDR is critical, considering the vulnerability of the local communities that depend on the environment for their livelihoods and the oil and gas activity that dominates the environmental challenges experienced (and indeed, which contribute to the climate change hazards to which the country has to adapt). Successive governments in Nigeria have embarked on several interventions to find solutions to environmental problems in the NDR, including the hydrological hazards it faces. One such strategy was the establishment of the Niger Delta Development Commission (NDDC); however, it has not delivered on its mandate for local communities [34,35]. The oil and gas activities that contribute to most of the environmental degradation, as well as corruption, inequality, and poverty, remained unchanged. The political interest of the Nigerian government remains a priority over the management of hydrological hazards and other related environmental degradation across the region [36]. The NDDC went further to note that efforts by Civil society to find solutions to environmental hazards in the Niger Delta have not been given a favourable response by the Federal government.

## 4. Methodology

Qualitative Document Analysis (QDA) was used to analyse national policy documents on the key risk sectors in the Niger Delta (Table 1). QDA offers a process of examining the content of policy documents that allows the identification of patterns, alongside the formation of codes and themes, and can be used across any geographical location. The specific QDA approach used here follows three steps in line with [37]: 1. setting criteria for the selection of national policies; 2. obtaining policy documents; and 3. analysis of policy documents. Each of these steps is detailed below.

**Table 1:** The linkages between National Policy Documents and Risk Sectors.

Sector	Policy document title	Year Published	Risk Sector
Environment	National Policy on Environment	2019	C, TC, I, HH, LS, FS, WS, P
Climate Change	National Climate Change Policy	2021	C, TC, I, HH, LS, FS, WS, P
Agricultural Plan	National Agricultural Technology Innovation Plan	2022	TC, FS, WS, HH
Forest Policy	National Forest Policy	2019	TC, C, FS, WS
Water Policy	National Water Resources Policy	2016	C, TC, FS, WS
Petroleum Policy	The National Petroleum Policy	2017	I

**Legend:** Low-lying coastal socio-ecological systems = C, Terrestrial and coastal ecosystems = TC, Physical infrastructure, networks, and services = I, Living standards = LS, Human health = HH, Food security = FS, Water security = WS, Peace and human mobility = P.

**Step 1): Setting the criteria for the selection of the policy documents.** The study applied a multi-step approach to identify relevant policy documents approved by the Nigerian government. First, we limited the scope to national policy documents approved from 2016 to 2022 across six key sectors (environment, climate change, water resources, agriculture, forest, and petroleum). The six selected policy documents are the keystone policies in each of the six departments that focus on the management of hydrological hazards (Table 1). A relevance score based on policy objectives was used to recognize how well a policy aligns its goals with the management of hydrological hazards in the NDR. Analysing these policies can highlight where the relevant institutions already align or where they can improve this in dealing with the effects of climate-related hazards.

**In step 2)** the national policy documents covering the sectors identified above were obtained on official government websites. In the case of the National Agricultural Technology Innovation Plan, the focal person on climate change for the Ministry of Agriculture was contacted, and the document was forwarded electronically as the policy was not publicly available online.
**Step 3) of the QDA: policy analysis.** The selected policies were analysed to answer the following questions:

a. What are the types of hydrological hazards and their drivers as noted in the policy documents?
b. What measures are outlined to reduce the risks specifically related to flooding, and
c. How aligned are they across the policies?

### 4.1 Keyword identification and processing

To address question a), keyword searches were performed in NVivo 12 using the text search query function. Hazard-related keywords such as sea level rise, river floods, high rainfall, storm surge, warming trend, and driver-related keywords, climate variability, urbanization, deforestation, industrialization, agriculture, infrastructure, population, across the six policy documents were drawn from the literature, encompassing the main hydrological hazards and their drivers.

A synonym/normalization dictionary (Table S1 in S1 File) was created to consolidate connected words into standardized categories (e.g., “flooded, flooding → Flood”). Stemming and lemmatization were enabled in NVivo. The frequency of each keyword was recorded for each policy.

### 4.2 Frequency threshold and binary coding

To avoid overemphasis on incidental mentions, a minimum frequency threshold of ≥2 mentions was applied. For each policy document:

- If a keyword occurred ≥2 times, it was coded as “1” (present)
- If it occurred <2 times, it was coded as “0” (absent).

Overall, policies were linked to the key risk sectors, to the specific drivers, and also to the specific hazards that they mentioned.

### 4.3 Identification of policy measures

To address question b), components in the policy documents’ aims, objectives, general statements, and actions were noted to obtain information about the measures to reduce hydrological hazards. Further, keywords, with the same process applied earlier (Table S1 in S1 File), were adopted, including adapt*, mitigat*, resilien*, vulnerab*, construction of levees along riverbanks, emission reduction, capacity building, financial capacity, and collaboration, were searched to understand the management of hydrological hazards across the policies. These words were chosen based on their significance in increasing resilience and reducing vulnerability to hydrological hazards in the identified sectors [38]. The appearance of these words across the policies and the context in which they were mentioned, especially as they relate to the management of hydrological hazards in the NDR, was noted. A matrix table was created to show the linkages between the policy documents and the frequency ranges with which the keywords appeared, with the following ranges: +=1–3, ++ = 4–6, +++ = 7–10, ++++ = more than 10 (Table 3). Thereafter, the analysis assessed the alignment across the policies, applying the keywords used for the measures to understand the policy strategies that are integrated into the management of hydrological hazards in the region. This used a weighted scoring system, adapted from [39]. Each policy was scored based on the policy that provided more measures to reduce hydrological hazards.

**Table 2:** The number of times each keyword appeared across the policy documents. SLR = Sea level rise, HR = High Rainfall, RF = River Flood, SS = Storm Surge, WT = Warming Trend, CV = Climate Variability, P = Population, D = Deforestation, U = Urbanisation, I = Industrialisation, A = Agriculture, IN= Infrastructure, ND = Niger Delta, C = Coast.

Policy	Keywords													
	Hydrological Hazards					Drivers							Regions	
	SRL	HR	RF	SS	WT	CV	P	D	U	I	A	IN	ND	C
Environment	3	8	32	8	7	50	22	55	150	19	52	84	14	35
Climate change	6	11	11	5	19	150	13	18	150	30	36	90	1	5
Agriculture	–	2	4	2	1	31	25	11	68	30	150	80	1	4
Forest	–	1	2	–	4	24	46	150	150	12	39	71	–	2
Water	–	6	6	–	–	3	7	–	68	6	17	50	1	1
Petroleum	–	–	–	–	–	–	14	1	93	26	–	80	46	3
Total	9	28	55	15	31	258	127	235	476	123	294	455	59	50

**Table 3:** Frequency threshold and binary coding.

	Policy Sectors					
Keywords	Environment	Climate change	Agriculture	Water	Forest	Petroleum
Sea level rise	1	1	0	0	0	0
High Rainfall	1	1	1	1	1	0
River Flood	1	1	1	1	1	0
Storm surges	1	1	1	0	0	0
Warming trend	1	1	1	0	1	0
Drivers of Hydrological Hazards						
Climate variability	1	1	1	1	1	0
Population	1	1	1	1	1	1
Deforestation	1	1	0	0	1	0
Urbanization	1	1	1	1	1	0
Industrialization	1	1	1	1	1	1
Agriculture	1	1	1	1	1	0
Infrastructure	1	1	1	1	1	1
Niger Delta	1	0	1	1	0	1
Coast	1	1	1	1	1	1

The last part of the question and the third stage of the QDA involved understanding how national policies align. Each policy document was reviewed systematically, identifying and interpreting statements relative to each other to reduce hydrological hazards and improve policy integration. The analysis highlighted the potential for connection and coordination with other policy sectors to lessen the impact of hydrological hazards across different areas. contained within the policy [37]. The QDA elucidated policy statements that set specific measures towards achieving climate resilience in the management of hydrological hazards, e.g., through forest and tree management. Each document was read multiple times to extract:

- Objectives, strategies, and actions related to climate resilience, flood control, and ecosystem management across the NDR.
- References to institutional collaboration, data sharing, and stakeholder engagement.
- Mentions of cross-sectoral linkages

Using deductive coding in line with [40], text segments were categorized under predefined analytical themes across the policies. We assess each policy along the following dimensions of alignment:

1. Hydrological hazard focus
2. Cross-sectoral integration
3. Alignment with climate goals

Coding was performed using NVivo 12 to ensure interpretive consistency across the six policy texts. To assess alignment strength, a three-level ordinal scoring system (High, Medium, Low) was applied across the three dimensions for each policy:

- High (3) = strong alignment; explicit cross-sectoral linkages and detailed hydrological hazard strategies.
- Medium (2) = moderate alignment; references to integration or resilience but limited operational detail.
- Low (1) = weak or absent alignment; siloed sectoral focus or conflicting objectives.

## 5. Results

This section is organized into three sections based on the research questions.

### 5.1 Hydrological hazards and their drivers as identified in the National Policy documents

Unsurprisingly, the environment and climate change policies mention the hydrological hazards more frequently than the other policies. The documents reference river floods and warming trends most frequently; river floods being mentioned 55 and warming trends mentioned 31 times across the whole set of policies. Similarly, the analysis found that the petroleum policy frequently mentioned the NDR because the oil and gas activity and their infrastructure are situated in the region Table 2. However, mention of the NDR is not linked to hydrological hazards; rather, the policy noted the adverse effects of environmental degradation in the region. The environment policy mentioned the NDR 14 times and the coast 35 times, and noted the effects of hydrological hazards and their linkages to the livelihoods of the communities that depend on the Delta. Climate change, agriculture, water, and forest policies mentioned the NDR the least, but refer to it in more general terms as “the coastal region”.

Table 3 displays the frequency threshold and binary coding of hydrological hazard-related keywords across six policy sectors in Nigeria. The analysis emphasizes how much specific environmental and climate drivers are integrated into sectoral policy documents. Conversely, petroleum shows limited or no involvement with hydrological risks. Agriculture, water, and forestry policies moderately include climate-related factors, especially climate variability and urbanization. The findings that emerge are a pattern of partial alignment but limited coherence [41]. While socio-economic drivers are generally acknowledged, the sectoral integration of specific hydrological hazards remains inconsistent throughout Nigeria’s policy landscape.

**Fig 3:**
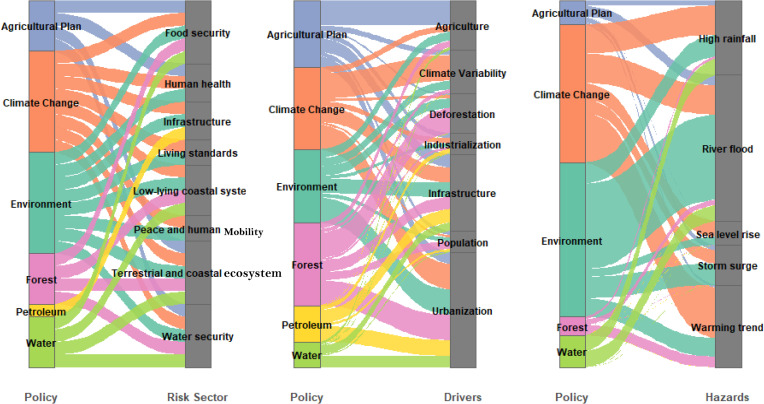
Weighted Policy Pathways Illustrating Linkages among a). Risk Sectors and the Policy Sector Coverage **b)** Policy Sector to Drivers and **c)** Policy Sector to Hazards. The flowlines represent the pathways through which specific policy areas align with risk-relevant issues across the study region.

**T**he policies were linked to the risk sectors, drivers, and hazards (Fig 3). The mapping of the drivers to the specific hazards shows the attribution of climate-related hazards to human influence, which has increased the frequency and severity of extreme climate events, including heat waves and heavy rainfall [42].

### 5.2 Measures to reduce hydrological risks

Table 4 presents a matrix of scores for policy approaches to hydrological hazard reduction ((identified using defined keywords in national documents**;** see Table S2 in S1 File).

**Table 4:** Matrix table showing the frequency of keywords across the six national policy documents. Legend: Adaptation = A, Mitigation = M, Resilience = R, Vulnerability = V, Flood Protection = FP, Emission Reduction = ER, Adaptive Capacity = AC, Financial Capacity = FC, and Collaboration = C.

Policy	A	M	R	V	FP	ER	AC	FC	C	Score
**Environment**	++	++	++	++	+	+	++	+++	++++	19
**Climate change**	++	++	+	++	+	+	+++	++	+++	17
**Agriculture**	–	+	+	–	+	+	+	+	+	7
**Forest**	++	+	+	+	+	+	++	+	+	11
**Water**		+	–	–	++		+	+	+	6
**Petroleum**		++	–	–	–	–	–	–	–	2

The Environment policy, followed by the Climate Change policy, offers the strongest support for managing hydrological hazards in the NDR. Specifically, the Environment policy emphasizes the need to “promote actions to protect and preserve land masses that help defend coastal areas and communities from the impacts of ocean waves” (Federal Ministry of Environment, 2016, p. 18). Similarly, the Climate Change policy recognizes the necessity of investing in “protective energy infrastructure to reduce loss and damage caused by climate-related extreme events” (Federal Ministry of Environment, 2021, p. 29). With a total keyword frequency score of 19, the Environment policy reflects a comprehensive commitment to enhancing resilience, protecting ecosystems, and building adaptive capacity along the country’s coastline.

Based on the matrix scores, the Agriculture, Forest, and Water policies provide moderate support for hydrological hazard management. These documents utilize a sector-specific approach rather than the integrated perspective found in the Environment and Climate Change policies. For instance:

1) Agriculture Policy: Notes the need to “reclaim degraded agricultural lands affected by soil erosion and flood to prevent their spread” (Federal Ministry of Agriculture and Rural Development, 2022, p. 63).
2) Forest Policy: Highlights the “regulation of water flow to control flood and soil erosion as well as sedimentation”.
3) Water Policy: Stresses the need to “improve real-time forecasting of hydrological phenomena to aid contingency plans for the reduction of the adverse effects of flood” (Federal Ministry of Water Resources, 2004, p. 10).

The Petroleum policy offers minimal support, focusing primarily on acknowledged environmental problems in the study region rather than specific hydrological management. It states that the “government is determined to address these environmental impacts through regulatory measures” (Federal Ministry of Petroleum Resources, p. 41), but specific measures for hydrological hazard management are absent.

Overall, these documents recognize the need for a “joined-up” approach to land management, such as avoiding construction in high-risk zones and reducing the conversion of forest land to agriculture. However, there is a critical need for sectors beyond Climate and Environment to adopt inter-sectoral innovations that enable “multiple wins” [43]. Such wins reduce environmental impacts while improving health equity and balancing adaptation with development through systematic changes across all governance levels [44].

**Fig 4:**
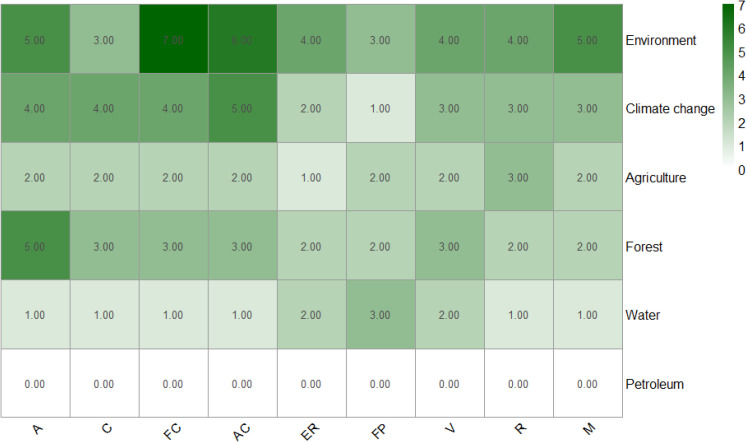
Policy sectors and their coverage based on the number of measures addressing each theme towards the management of hydrological hazards across the NDR. Legend: Adaptation = A, Mitigation = M, Resilience = R, Vulnerability = V, Flood Protection = FP, Emission Reduction = ER, Adaptive Capacity = AC, Financial Capacity = FC, and Collaboration = C.

Fig 4 illustrates the thematic coverage of these policies. While Environment and Climate Change policies are integrated and climate-responsive, others remain sector-specific. The clustering of Environment and Climate Change policies suggests an opportunity for joint leadership, whereas the Petroleum policy represents a significant gap in the energy sector’s climate considerations.

### 5.3 Alignment across the policies

The alignment analysis across the six national policies reveals varied degrees of coherence in addressing hydrological hazards in the NDR. Most measures are linked to climate adaptation, resilience building, vulnerability reduction, and flood protection. These policies highlight cross-cutting issues, such as gender equality and green recovery, which provide a foundation for inter-sectoral collaboration (see the measures identified through the defined keywords in the national policy documents Table S2 in S1 File).

The Environment and Climate Change policies exhibit strong thematic alignment in adaptation and resilience. For example, the Environment policy suggests implementing “mitigation measures to address climate change and its impact on freshwater and wetland ecosystems” (Federal Ministry of Environment, 2016, p. 16), while the Climate Change policy aims to “facilitate public and private sector investments in capacity building” (Federal Ministry of Environment, 2021, p. 40).

The Agriculture and Water policies show moderate linkage through shared concerns regarding water management and early warning systems. The Agriculture policy emphasizes “the timely provision of weather and climate information to farmers,” while the Water policy hinges management on “an effective water resources assessment program” (Federal Ministry of Water Resources, 2016, p. 10). The Forest policy aligns conceptually with the Environment policy regarding ecosystem-based adaptation, particularly through mangrove restoration, but remains institutionally disconnected. The Forest policy emphasizes that the country should “encourage collaborative partnership with rural communities for the sustainable management of forest resources to ensure the supply of goods and services from the forest for the present and future generations” (The Federal Ministry of Environment, 2019, p. 31). The analysis equally highlighted the potential for connectivity across the policies (Table 5).

**Table 5:** Overview of how the six national policies document connectivity to manage hydrological hazards, noting the interconnections and combined methods needed across different sectors.

Sector	Policy Focus	Alignment with Hydrological Management
Environment	Coastal Zone Management Plan, Environmental Impact Assessment	Implement integrated coastal zone management to protect coastal ecosystems and prevent increased hydrological
Climate change	Climate adaptation strategies targeted at the reduction of greenhouse gas emissions	Develop adaptation and mitigation plans to minimise and address the projected climate-related hazards, especially hydrological hazards, in the coastal area
Agriculture	Climate-smart agriculture and sustainable water use in farming	Implement buffer zones to protect farmland from floods.
Forest	Mangrove restoration	Restore and protect mangroves as natural barriers against storm surges and coastal erosion
Water	Integrated water resources management (IWRM)	Effective IWRM plans that account for the impacts of climate change on water availability
Petroleum	Spill prevention and response plan	Implement rigorous spill prevention and response plans to protect coastal waters and the environment

Integrating these measures is essential for the harmonized management of hydrological hazards. Effective management requires a unified approach that leverages collaboration, addresses trade-offs, and encourages data sharing. The Petroleum policy, while acknowledging environmental problems in the NDR, demonstrates the weakest integration with the broader environmental framework. The alignment of national policies is vital for managing hydrological hazards in the NDR. Currently, this operational potential appears weak. Inadequacies in alignment are often attributed to poor coordination across national, state, and local government levels (Aigbe et al., 2023). Table 6 summarizes these alignment strengths, underscoring that while cross-cutting objectives are recognized in principle, their application remains fragmented.

**Table 6:** Summarizes the alignment matrix across the policy documents.

Policy Sector	Hydrological hazard focus	Cross-sectoral integration	Alignment with climate goals	Implementation coherence	Alignment strength
Environment	Strong on adaptation, ecosystem resilience	Integrates with all sectors	Very High	High	High
Climate change	Central policy for mitigation & adaptation	Integrates with all sectors	Very High	High	High
Agriculture	Mentions weather/climate info; weak on flood control	Sector specific	Moderate	Medium	Medium
Water	Focused on flood control & watershed protection	Sector specific	Low	Medium	Medium
Forest	Biodiversity & ecosystem-based adaptation	Linked to Environment	Moderate	Medium	Low
Petroleum	Focused on emission reduction & energy efficiency	Poor integration with others	Low	Weak	Low

Overall, the findings underscore that while cross-cutting objectives are recognized in principle, application remains fragmented. Strengthening institutional coordination, shared data frameworks, and integrated policy instruments across these sectors is essential for effective hydrological hazard management in the NDR.

## 6. Discussion

### 6.1 Cross-sectoral policy alignment and hydrological hazard focus

This study evaluated six national policy documents to assess their alignment and coherence in managing hydrological hazards within the NDR. The results reveal substantial variations in policy emphasis and the strength of integration. While the Environment and Climate Change policies directly address hydrological hazards and their underlying climatic drivers, the Agriculture, Water, and Forest policies largely operate within sectoral silos. In these latter documents, hydrological hazards are treated as indirect consequences of environmental degradation rather than explicit priorities. This confirms the horizontal coordination challenges frequently identified in policy research. These findings support existing policy integration frameworks [45–47], which emphasize the necessity of mainstreaming cross-cutting risks such as flooding and sea-level rise into sectoral policies. Within the Nigerian context, the Environment and Climate Change policies constitute the core alignment cluster, whereas Agriculture, Water, and Forest form a secondary, partially aligned cluster. The Petroleum policy remains peripherally aligned (Fig 4 and Table 5). This structural imbalance significantly limits Nigeria’s overall adaptive capacity to manage hydrological hazards in the NDR.

### 6.2 Policy measures and sectoral priorities

Across the six policies, hydrological hazards are primarily recognized through the lenses of climate change adaptation, ecosystem resilience, and land degradation control. The Environment and Climate Change policies offer the most comprehensive measures, including the development of mitigation strategies, the implementation of early warning systems, and the restoration of mangroves and wetlands to buffer against storm surges and flooding. Such measures have direct implications for reducing flood vulnerability and enhancing ecosystem services in the NDR.

In contrast, the Agriculture, Water, and Forest sectors exhibit fragmented measures. For instance, while the Agriculture policy promotes weather and climate information services, it lacks robust integration with water and land management frameworks. Similarly, the Water policy prioritizes watershed protection but remains weakly linked to forest conservation and broader climate action. This pattern indicates horizontal misalignment, a recurrent challenge in environmental policy integration literature [46,47].

### 6.3 The petroleum policy and political economy constraints

The petroleum policy stands out for its very limited consideration of hydrological hazards, despite the sector’s well-documented role in shaping environmental vulnerability in the NDR risks [48]. This omission can be understood through a political economy perspective. Nigeria’s dependence on petroleum revenue has fostered a rentier state dynamic in which short-term fiscal priorities dominate, while long-term sustainability and hazard management are sidelined [49]. The sector is heavily influenced by powerful interest groups, including multinational corporations and domestic elites, who often resist stricter environmental regulations that could constrain production. This gap highlights how powerful sectoral interests can weaken integration, a common barrier, especially in developing-country contexts [50,51]. Furthermore, Nigeria’s federal governance structure centralizes control of petroleum resources in Abuja, restricting the influence of communities most affected by hydrological hazards. This reinforces the marginalization of hazard management in petroleum policy, highlighting the role of structural power inequalities and vested interests in shaping policy priorities, such asymmetric power relations constrain cross-sectoral alignment and weaken hazard management across the NDR.

### 6.4 Multi-level governance and coordination challenges

The study highlights that multi-level governance deficits, both horizontal across ministries and vertical across federal, state, and local levels, are central to Nigeria’s policy misalignment. Although Nigeria’s commitments under the UNFCCC emphasize climate resilience, translating these ambitions into coordinated, multi-sectoral actions remain limited. The federal system of governance concentrates decision-making power in Abuja, thereby constraining sub-national agencies that are closest to the communities most affected by hydrological hazards. This top-down structure undermines effective implementation and inter-agency communication, echoing similar governance challenges observed in South Africa and Ghana [52,53]. Addressing these challenges requires stronger vertical integration frameworks, connecting national policies with state and local institutions to improve responsiveness and implementation capacity [54,55].

### 6.5 Comparative and regional perspectives

Nigeria’s policy landscape reflects broader African challenges in climate and hydrological hazard policy integration. Examples from Kenya, Ghana, and South Africa show that although progress has been made in mainstreaming adaptation into agriculture and water policies, implementation barriers and inter-agency silos persist [51]. In contrast, countries with more established climate legislative frameworks, such as those in Europe and the Asia Pacific, demonstrate more effective cross-sectoral coordination and enforcement, supported by stronger institutional capacity and technological innovation [56]. African countries, on the other hand, are faced with inequalities in national economies, technological advancements, and industrial structures within the region [57]. Contextualize findings within the literature, showing how fragmented institutional architecture and low vertical coordination undermine adaptive hydrological governance in coastal systems, consistent with [58,59], but with novel insights for the Niger Delta context.

Nigeria’s comparatively modest performance on continental climate indices (55.8%, compared to Morocco’s 68%) underscores the urgency of strengthening institutional coherence. Improved coordination across ministries, coupled with local-level engagement, could transform fragmented governance into a resilient, adaptive policy regime for hydrological hazard management.

### 6.6 Toward integrated hydrological governance

Overall, the findings demonstrate that while the Environment and Climate Change policies provide a strong foundation for hydrological hazard management, integration across other sectors remains partial and inconsistent. The observed fragmentation reflects deeper structural issues, limited institutional capacity, overlapping mandates, and insufficient communication mechanisms [60]. By implementing national policies effectively and collaboratively across government levels, Nigeria can move beyond a fragmented, top-down approach towards adaptive, inclusive governance that benefits the people of the NDR.

### 6.7 Recommendations

Establish a Cross-Sectoral Coordination Mechanism. Nigeria should establish a National Hydrological Hazard Management Task Force comprising representatives from key ministries (Environment, Climate Change, Agriculture, Water Resources, and Petroleum), state governments within the NDR, civil society, and community groups. Previous research advocates for such multi-sectoral platforms in Africa to address water-related climate risks effectively [61]. This task force would serve as a permanent body to harmonize policy objectives, monitor implementation, and ensure that hazard management measures are integrated across all sectors. Similar structures, such as the UK’s Transition Plan Taskforce’s Adaptation Working Group, successfully oversee national adaptation strategies [62].

Implement Policy Pilot Zones in the Niger Delta. Place-based policies have been widely adopted globally to support regional development [63,64]. Policymakers should designate specific Pilot Zones (e.g., coastal wetlands and mangrove belts) in the NDR to test integrated policy interventions. In these zones, agricultural, forestry, water, and petroleum policies would be applied in a coordinated manner, with local communities actively engaged in design and monitoring. These pilots would demonstrate the feasibility of cross-sectoral adaptation strategies and provide a scalable evidence base for the rest of the region.

Legislative Amendments for Policy Alignment: Sectoral laws, including the Petroleum Act, Water Resources Act, and National Agricultural Policy, should be amended to explicitly mandate the consideration of hydrological hazards and climate risks. For instance, Environmental Impact Assessments (EIAs) for petroleum projects should be legally required to incorporate flood risk projections, while agricultural and forestry laws should mandate sustainable land-use practices to reduce hazard exposure. There is a clear call for mainstreaming such adaptation into sectoral legislation in Africa to reduce fragmented responses. [65]. Legal provisions would create binding obligations, reducing the current reliance on discretionary alignment.

Strengthening Multi-Level Governance. Subnational participation must be institutionalized by mandating the representation of the Niger Delta states in federal climate and hazard management committees. This shift would reduce the top-down bias of the current system, ensuring that local knowledge and grassroots needs are integrated into national decision-making processes.

## 7. Conclusion

This study demonstrates that while Nigeria’s national policy documents acknowledge hydrological hazards and their primary drivers, inter-sectoral alignment remains limited, particularly within the petroleum, agriculture, and forestry domains. This lack of coherence risks fragmented action, duplication of efforts, and significant gaps in addressing the critical drivers of flood risk in the NDR.

The analysed policies characterize hydrological hazards as water-related phenomena that pose severe risks to life, property, infrastructure, and the environment in the low-lying NDR. This vulnerability is exacerbated by the region’s low adaptive capacity. Key drivers identified across the policies include agricultural expansion, mangrove deforestation, population growth, and climate variability. While the policies outline essential management steps such as community-based mitigation, prompt response initiatives, and capacity building for forecasting, the government’s role in enforcing these policies and creating an enabling environment is paramount for building resilience.

Strengthening horizontal and vertical policy integration is vital for the region’s climate resilience. Conflicting objectives can result in wasted resources, institutional “buck-passing,” and weak enforcement at subnational levels. To bridge the gap between coherence analysis and practical application, future research should evaluate implementation outcomes using specific metrics, such as policy execution rates, the frequency of cross-sectoral collaborations, and measurable reductions in hydrological disaster losses. Such indicators will provide the necessary evidence to determine whether enhanced policy alignment leads to more effective flood risk governance and sustainable development in the Niger Delta.

## Supporting information

S1 File Synonym/normalization dictionary and measures identified on the defined keywords across the national policy documents sectors. (PDF)
