# Supplementary material for: Understanding policy alignment in addressing hydrological hazards in the Niger Delta region, Nigeria
Source: PLoS One. 2026 Mar 25;21(3):e0345583. doi: 10.1371/journal.pone.0345583 (PMC13016295; doi:10.1371/journal.pone.0345583)
Supplement: S1 File — (PDF) [file pone.0345583.s001.pdf]

S1 Supporting Information.

Table S1

**Standard Keyword**	**Terms Included**
River Flood (RF)	Flood, flooding, river flood, flood disaster
Storm Surge (SS)	Storm surge, coastal surge, tidal flooding
Sea Level Rise (SLR)	Sea level rise, coastal inundation
High Rainfall (HR)	High rainfall, heavy precipitation, intense rainfall
Warming Trend (WT)	Warming, temperature rise, heat trend
Climate Variability (CV)	Climate variability, seasonal anomalies
Population	inhabitants, residents, populace, citizenry, community
Deforestation	Tree-clearing, forest clearing, logging, clear-cutting
Urbanisation	Urban development, city growth, suburbanization,
Industrialisation	Manufacturing growth, mechanization, factory growth
Agriculture:	Farming, cultivation, husbandry, agribusiness, food production
Infrastructure	Facilities, utilities, services, public works, transport networks, and essential services
Niger Delta	Low-lying
Coast	Seaboard, shore, shoreline, seaside, littoral (an adjective, but often used for the coastal
**Measures identified**	
Adaptation	Adjustment, acclimation, and Confirmation
Mitigation	Ease, Lessen, Lighten, Moderate, Subdue
Resilience	Strength, Persistence, Perseverance, and Potency
Vulnerability	Susceptibility, weakness, exposure, and defencelessness
Flood protection	Flood control, Flood defence, Flood prevention, Flood management, and Flood resilience
Emission reduction	Abatement, Decarbonization, Carbon removal, Curtailment, and Tackling emissions
Adaptive capacity	Adjustability, Adaptability, Changeability, and Flexibility

Financial capacity	Financial resources, Fiscal capacity, and Monetary strength
Collaboration	Cooperation, Partnership, and Coordination

Table S2

The acronyms of the policy sector are represented as follows: Environment =1, Climate change =2, Agriculture=3, Forest=4, Water=5, and Petroleum=6

Policy	Adaptation	Mitigation	Resilience	Vulnerability	Flood Protection	Emission Reduction	Adaptive capacity	Financial Capacity	International collaboration
1	-Raise awareness of climate change adaptation opportunities among various stakeholders at all levels<br><br>-Extensive adaptation measures that are necessary to reduce vulnerability to future climate change<br><br>-Protect wetlands, riverbanks, hilltops, and slopes from unsustainable practices to prevent soil erosion and environmental degradation.<br><br>- Ensure that all development activities conform to	-Raise awareness of climate change mitigation opportunities among various stakeholders<br><br>-Extensive mitigation measures that are necessary to reduce vulnerability to future climate change are put in place<br><br>-implement mitigation measures to address climate change and its impact on freshwater and wetland ecosystems.<br><br>-Develop flood forecasting, prediction, and early warning systems for major river systems in the country	-Promote the development and implementation of environmental governance to build resilient<br><br>-implement a harmonized integrated Ecological Master Plan (EMP) as well as a Coastal Zone Management Action Plan<br><br>-Development of an aggressive management and enforcement programme to protect and maintain the quality of the nation's soil resources from floods and erosion.<br><br>-Encourage public-private sector partnership in the sustainable management of natural resources	-Implement the West Africa Coastal Areas (WACA) programme to reduce the country's vulnerability to coastal erosion, flooding, and sedimentation problems.<br><br>-Coordinate the roles of all agencies interfacing with the management of coastal and marine ecosystems for optimum resource protection.<br><br>-Implement the Nigeria Erosion and Watershed Management Programme (NEWMAP) to reduce the country's	-Control physical growths including reclamation of wetlands for socio-economic development<br><br>-Promote actions to protect and preserve land masses that help defend coastal areas and communities from the impacts of ocean waves<br><br>-Ensure that developmental activities within the freshwater and wetland ecosystems conform to EIA process and procedures.	-Implementation of UNFCCC, including but not limited to the implementation of the NDCs and the Paris Agreement<br><br>-Formulate and implement innovative strategies to increase forest and tree cover to at least 25% of the total land area (in line with the FAO standard).<br><br>- Develop and implement measures to control emissions from motor vehicles, power plants, and local industrial and commercial processes.<br><br>-	-Build capacity, share knowledge, and ensure community participation in the plans and management of coastal and marine ecosystems<br><br>-Support and promote research and cross-sectoral capacity enhancement in conserving, protecting, and managing marine and coastal resources.<br><br>-Promote integrated watershed management and alternative livelihood opportunities to enhance community participation	-Develop and implement an Integrated Financial Strategy for Climate Change Response<br><br>-Participate in multilateral negotiations and fulfill agreed obligations including those of finance.<br><br>-Provide, continuously, necessary and desirable assistance to exporters on environmentally friendly product development and financing	-Maintain a roster of multilateral agreements on the environment entered into by Nigeria.<br><br>-Develop, implement, and strengthen programmes for international scientific collaboration, sharing of information, and technology transfer<br><br>-Develop and implement delegation instruments to provide a framework for cooperative and collaborative management between the Federal Ministry of Environment, other Ministries, Departments, and Agencies (MDAs).<br><br>-collaboration with the Federal Ministries of Civil Aviation (Nigerian Meteorological Services – NIMET) and Health, as well as other relevant Agencies.

Policy	Adaptation	Mitigation	Resilience	Vulnerability	Flood Protection	Emission Reduction	Adaptive capacity	Financial Capacity	International collaboration
	Strategic Environmental Assessment and Environmental Impact Assessment.	-Develop and implement community-based flood mitigations and prompt response measures and initiatives to minimize the impacts of flooding.	for poverty reduction	vulnerability to land degradation and strengthen the national enabling environment for effective implementation of erosion and watershed management.<br><br>-Prepare comprehensive hazard maps and vulnerability analysis for the country			-Build capacity for flood forecasting and monitoring.<br><br>-Strengthen capacity and infrastructure to monitor environmental (air, water and noise) pollution<br><br>-Strengthen national climate change institutional structure and governance to include active participation by the States and the Local Governments.<br><br>-Strengthen national climate change institutional structure and governance to include active participation by the States and the Local Governments.		-Building partnerships among all stakeholders, including government at all levels, international institutions and governments, non-governmental agencies, and communities on environmental matters.<br><br>-.Implement the existing international agreements on freshwater resources and promote partnership and cooperation at regional and global levels, particularly in the development and management of shared rivers, lakes, and wetlands.<br><br>-Intensify international and regional cooperation and partnership arrangements in the management of shared Land and water resources<br><br>-Enhance public-private partnerships in environmental management.<br><br>-Develop and implement a Strategy on Partnership and

Policy	Adaptation	Mitigation	Resilience	Vulnerability	Flood Protection	Emission Reduction	Adaptive capacity	Financial Capacity	International collaboration
									Stakeholder involvement to enhance environmental management.<br>-
2	Increased awareness of climate change impacts and adaptation measures.<br><br>- Enhance adaptation capacity whilst also providing for effective implementation and enforcement procedures.<br><br>-- Enhance forest capacity for adaptation by reducing ecosystem vulnerability and also reducing exposure of the ecosystems to extreme events<br><br>-Support main settlements to develop and undertake ambitious climate change adaptation actions.	Increased awareness of climate change impacts and mitigation measures.<br><br>- Identify the mitigation options that reduce the risk of longer-term climate change<br><br>- Implement mitigation measures that will promote low carbon as well as sustainable and high economic growth<br><br>- Identify the mitigation options that reduce the risk of longer-term climate change<br><br>- Invest in early warning systems, including reliable and timely weather and hydrometeorologi cal observations	Fortifying power plants close to coastlines prone to flooding and erosion can safeguard resilience.<br><br>- Strengthen integrated water resources management (IWRM) for multi-layered development of the nation's water resources infrastructure<br><br>-Low-emission resilient development pathway and help mainstream climate action into sectoral policies	Implementing appropriate strategies and actions to reduce the vulnerability of Nigerians to the impacts of climate change across all sectors<br><br>- Developing and implementing appropriate strategies and actions to reduce the vulnerability of Nigerians to the impacts of climate change across all sectors<br><br>- Develop robust projections in terms of climate change impacts for the formulation of appropriate policies towards reducing vulnerability	Invest in protective energy infrastructure to reduce loss and damage caused by climate-related extreme events	Implementing adaptation and mitigation measures that promote low-carbon development<br><br>- Inclusion of a longer-term vision for low-emission climate resilient development	Strengthen capacity for smart water management<br><br>- Enhance forest capacity for adaptation by reducing ecosystem vulnerability and also reducing exposure of the ecosystems to extreme events<br><br>- Enhance understanding of the overall gender dimensions of climate change<br><br>- Capacity building, training and institutional strengthening<br><br>-Strengthen capacity for smart water management<br><br>-Improve understanding of the climate system and its drivers	the role and operation of innovative financing instruments like the Green Bonds in emissions reduction and climate change adaptation<br><br>-financing and monitoring of climate change initiatives and programmes in the country.<br><br>-Enhancing national capacity to mobilize international and national resources, both technical and financial, for investment in climate change<br><br>-Facilitate an enabling environment for enhanced public and private sector participation and financial investments to	Promote international research and collaboration<br><br>-Strengthen the capacity of the Department of Climate Change in collaboration with other relevant technical Departments in the Federal Ministry of Environment to play the oversight role of monitoring the implementation of the policy<br><br>-Establish effective and sustainable Public Private Partnership for the provision of climate -smart water supply and sanitation facilities and infrastructure<br><br>-Facilitate international partnerships to reinforce cooperation for climate resilience in the sector

Policy	Adaptation	Mitigation	Resilience	Vulnerability	Flood Protection	Emission Reduction	Adaptive capacity	Financial Capacity	International collaboration
		combined with forecast model		-Improve understanding of climate change impacts and vulnerability;			-Strengthen national and sub-national government capabilities of mainstreaming climate actions and implementation, including linkages	achieve adaptation at scale<br><br>-Increase access to adaptation finance through economic incentives and value chain initiatives<br><br>-Facilitate the establishment of the National Climate Change Trust Fund	
3		Ensure effective risk mitigation for investors, smallholder farmers and other stakeholders	Increase the resilience of vulnerable people's livelihoods to threats and crises and contribute to the reduction of food insecurity and malnutrition<br><br>-Increased resilience through digital and climate-smart agriculture, towards promoting agricultural value chains and investments.<br><br>-Build resilience and social protection nets through food and nutrition systems		Improve Soil Conservation (Erosion Control) structures	Boost productivity and reduced methane gas and other GHGs emission.	Facilitate the deployment of water harvesting and run-off flows technologies and techniques to enhance irrigation	Adoption of insurance scheme to support environmental Risks -Climate change - Natural hazards (flood, outbreak of pests and diseases)	Drastically reduce the menace of incessant flooding using regional and national collaborative (regulatory and investment)<br><br>-Promotion of publicprivate partnership for the conduct of adaptive research,<br>-Drastically reduce the menace of incessant flooding using regional and national collaborative (regulatory and investment)

Policy	Adaptation	Mitigation	Resilience	Vulnerability	Flood Protection	Emission Reduction	Adaptive capacity	Financial Capacity	International collaboration
			for vulnerable groups;						
4	Sustain and enhance the role of the forestry sector in climate change adaptation, and stakeholder's access to benefits.<br><br>-The sector has a critical role in climate change adaptation at the local, national and global levels.<br><br>-Enhance adaptation of people and ecosystems to climate change and climate variability<br><br>-Assist the rural poor to better adapt to climatic change	Sustain and enhance the role of the forestry sector in climate change mitigation and stakeholder's access to benefits.<br><br>-The sector has a critical role in climate change mitigation at the local, national and global levels.<br><br>-Forests contribute significantly to climate change mitigation through their carbon sink and carbon storage functions.	Support sustainable agro-forestry practices in off-reserved areas to enhance food and nutrition security, climate change resilience,	They play an essential role in reducing vulnerabilities	Regulation of water flow – that is maintenance of dry season flows and flood control<br><br>-Control of soil erosion and sedimentation and enhancement of productivity.	. Strategies For Climate Change, Reducing Emission From Deforestation and Forest Degradation (REDD+), Carbon Credit Trading and Other Similar Mechanisms	Build capacity and develop institutions for the effective stakeholder participation in forest resources management, research and development at all levels.<br><br>-To ensure the capacity of forests to protect watersheds, buffer zones around rivers and hills, sensitive ecosystems services, prevention of water and wind erosion, siltation of water courses and support for carbon sequestration.	Create an enabling environment for accessing global opportunities for forestry financing and development.	Provide economic, social, and financial incentives to encourage commercial tree growers and sustain collaborative partnership<br>-Encourage and strengthen networking among various research institutions, national and international.<br><br>-Sustainable Management of the Forest Estate in collaboration with the Local Government, NGOs, Communities, and civil society organisations
5		This assessment also includes water utilisation and activities for mitigating water-related disasters such as floods		The management of hydrological risks and vulnerabilities shall be hinged on an effective water resources	To improve real-time forecasting of hydrological phenomena that will aid contingency plans for the reduction of the		Research shall strengthen local capacities and help finding cost effective and feasible solutions, intended to promote best practices for	Encourage financial institutions to provide Loans to the water service sector.	Establish comprehensive monitoring system for water resources in collaboration with coriparian in all its boundary basins for essential data collection with a uniform format

Policy	Adaptation	Mitigation	Resilience	Vulnerability	Flood Protection	Emission Reduction	Adaptive capacity	Financial Capacity	International collaboration
				assessment program.<br><br>-Support the regional agencies' activities, meet its own commitment and exert influence to ensure protection of her interest as a vulnerable downstream riparian state.	adverse effects of drought and flood.<br><br>-Abstraction, return/recharge , and apportionment, effective flood damage reduction<br><br>-The management of hydrological risks and vulnerabilities shall be hinged on an effective water resources assessment program.<br><br>Support the regional agencies' activities, meet its own commitment and exert influence to ensure protection of her interest as a vulnerable downstream riparian state.		technical and environmental requirements.<br><br>-Technical and financial capacity building to efficiently manage water delivery system.		to be collated, analyzed and shared
6		There is a need for increased cooperation and coordination to mitigate the		Vulnerable groups need to be protected					

Policy	Adaptation	Mitigation	Resilience	Vulnerability	Flood Protection	Emission Reduction	Adaptive capacity	Financial Capacity	International collaboration
		impacts of oil industry activity among all stakeholders<br><br>-Mitigating social consequences of natural resource exploitation – best practice community participation.<br><br>-polluters pay the full costs of mitigating any damage to the environment or communities.<br><br>Lack of real mitigation of the damage simply lead to more resentment and ultimately more militancy.<br><br>-An investigation will be carried out into these two areas of risk, to identify the level of risk and recommended mitigations							
